# Skin-Inspired Flexoelectret Touch Sensor for Self-Powered and Self-Calibrated Tactile Imaging

**DOI:** 10.34133/research.1419

**Published:** 2026-08-31

**Authors:** Rui Ge, Qiuhong Yu, Yongkang Zhang, Yafeng Cai, Shuhai Liu, Weimin Liu, Jun Zhang, Yong Qin

**Affiliations:** ^1^ College of Information and Communication Engineering/College of Integrated Circuits, Harbin Engineering University, Harbin, Heilongjiang 150001, China.; ^2^ Institute of Nanoscience and Nanotechnology, School of Materials and Energy, Lanzhou University, Lanzhou, Gansu 730000, China.; ^3^ Henan Key Laboratory of Photoelectric Energy Storage Materials and Applications, School of Physics and Engineering, Henan University of Science and Technology, Luoyang, Henan 471000, China.; ^4^ State Key Laboratory of Solid Lubrication, Lanzhou Institute of Chemical Physics, Chinese Academy of Sciences, Lanzhou 730000, China.; ^5^ State Key Laboratory of Environment Characteristics and Effects for Near-space, School of Interdisciplinary Sciences, Beijing Institute of Technology, Beijing 100081, China.

## Abstract

While critical for human–machine interfaces, on-skin touch sensors struggle to replicate human skin’s curved deformation mechanics and achieve simultaneous high sensitivity, linearity, robustness, and stability. Existing capacitive, piezoresistive, piezoelectric, or triboelectric mechanisms cannot effectively harness touch-induced curved indentation. We introduce a skin-inspired flexoelectret touch sensor that exploits flexoelectricity—converting strain gradients into electricity—to mimic skin’s curved deformation geometry. This enables self-powered, self-calibrated tactile imaging through a novel mechanism establishing linear correlation between inhomogeneous deformation and electrical signal. The sensor achieves high sensitivity (the highest sensitivity up to ~835 mV/N), good linearity, exceptional robustness (>80,000 s continuous operation; >8,000 cycles), and immunity to temperature fluctuations, ultraviolet light illumination, and humidity interference. A 7 × 7-pixel array demonstrates shape-adaptive and touch-trajectory imaging capabilities. This technology unlocks transformative potential for wearable electronics and advanced human–machine interactions.

## Introduction

Among multiple sense modalities such as touch, vision, and audio, the sense of touch is particularly important, as it enables body awareness—the presence and occupancy of space. It involves complex physical interaction and plays a fundamental role in estimating contact properties of real-world objects such as press strength, spatial position, shape, and touch trajectory. Investigating such interaction is important in behavioral studies, healthcare, human–machine interfaces, and robotics applications. A lot of effort has been made to develop touch sensing technology that can sensitively and robustly convert external/internal mechanical stimuli into electrical signals. Breakthroughs in material design and device fabrication have led to the development of on-skin sensors with new working principles and structural designs. Notable examples include piezoelectric biomaterials [1] and transducers [2] for biological vibration detection, piezotronic transistors for tactile imaging [3–5], piezoionic skin for mechanoreceptors [6], and adhesive semiconductors for biointerfaces [7]. These state-of-the-art advancements have broadened the capabilities of touch sensing technology [8–17], enabling it to offer more comprehensive functionality and higher adaptability, and thus to closely mimic the real skin.

Over the years, touch sensing based on capacitive and piezoresistive effect has been extensively investigated and successfully applied in medical and industrial fields, due to the well-understood mechanisms and excellent stability. Touch sensing based on piezoelectric, triboelectric, and pyroelectric effect induced by polar symmetry can utilize the stimuli-induced polarization to generate electricity and thus to sense the stimuli by evaluating the strength of electrical signals. This is the so-called “self-powered” feature and has drawn tremendous interest with the trend of miniaturization and multi-functional integration of on-skin sensing [1,18–20]. This kind of self-powered sensing does not need the external power source but relies on the energy of applied stimuli, and has triggered plenty of new applications such as self-powered tactile imaging, self-powered cardiovascular electronics, and self-powered electronic tongue [2,21,22].

**Fig. 1.**
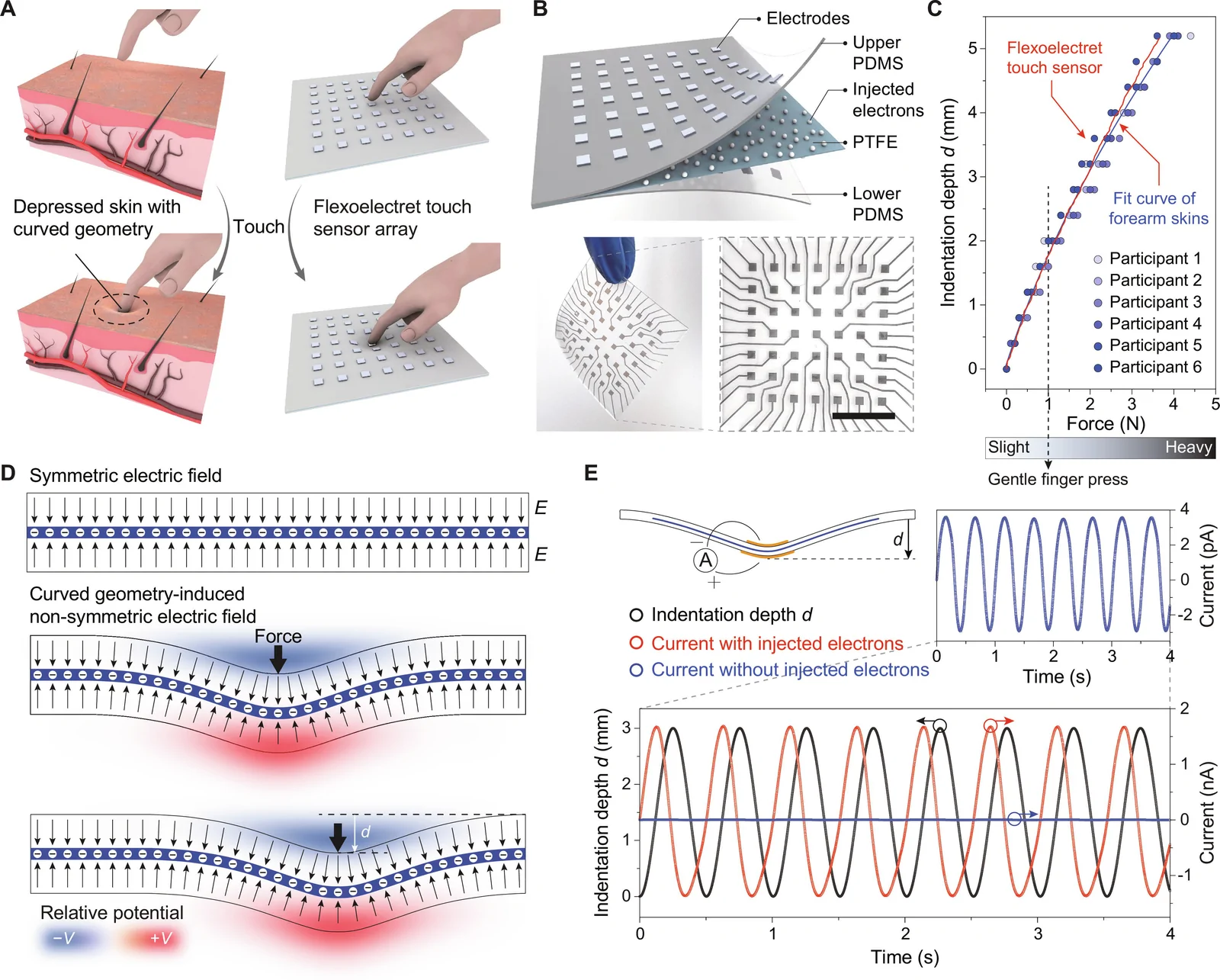
Design and mechanism of the skin-inspired flexoelectret touch sensor for touch sensing. (A) Schematic representation comparing the depressed skin and the flexoelectret touch sensor with curved geometry. (B) Illustration showing the design concept for the flexoelectret touch sensor prototype (top) and the corresponding optical images (bottom). The inset in the dotted box depicts the fabricated 7 × 7-pixel flexoelectret touch sensor array with pitch of 7 mm (scale bar, 20 mm). (C) Indentation depth *d* as a function of the depressed force measured on the forearm skins of 6 participants (blue) and the flexoelectret touch sensor with a thickness of 2.0 mm (red). (D) Transduction mechanism of the flexoelectret touch sensor based on the curved geometry-induced nonsymmetric electric field *E*. (E) Waveforms of output current under a sinusoidal indentation depth (black) generated by the flexoelectret touch sensor with (red) and without (blue) injected electrons.

In recent years, flexoelectricity has begun to attract worldwide attention for its capacity to convert inhomogeneous deformation, such as bending, into flexoelectric polarization and thus electricity in materials of any symmetry [23]. It is a special effect that can establish a linear relationship between the applied strain gradient from curved geometry and the generated electricity [24,25]. This unique mechanism makes it eminently suitable for self-powered sensing of flexible electronics [26–29]. Coincidentally and excitingly, the real skin will be accompanied by a curved geometry when feeling the touch (Fig. 1A), which is very similar to the case of introducing a strain gradient and generating flexoelectricity on the surface of material. This similarity between them highly promotes us to consider whether the flexoelectric effect can be used for self-powered touch sensing. If realized, it will realistically imitate the indentation from curved geometry of the real skin to sense the touch. Moreover, considering that the depressed skin with curved geometry is a region rather than a single point (touch point), we may also use the sensing information on the adjacent region around the touch point to precisely calibrate and localize the touch point. In other words, flexoelectricity provides a possibility for self-calibrated touch sensing.

As for such touch sensing based on flexoelectricity, a number of desirable properties [30] must also be taken into consideration, including high sensitivity, high linearity, high robustness, and even high anti-interference capacity. First, the sensing sensitivity should be maximized to enhance signal resolution and simplify readout electronics. Second, a linear sensing response (a common feature of conventional mechanical sensors) is indispensable to reduce device complexity and facilitate calibration. Third, the robustness of withstanding long-time continuous testing or a large number of mechanical cycles (e.g., >500) to ensure reliable touch sensing is essential for practical applications. Fourth, the anti-interference capability of keeping insensitive to environmental noises like temperature fluctuations or light illumination is also advantageous for providing intrinsic disturbance rejection and reducing the complexity of closed-loop control system. A combination of all these desired characteristics in one device is still challenging [30,31].

Herein, to mimic the depressed skin with curved geometry during touch (left, Fig. 1A), we proposed and developed a skin-inspired flexoelectret touch sensor for self-powered and self-calibrated tactile imaging (right, Fig. 1A). It possesses 2 advancements: one is self-powering feature that utilizes the touch-induced curved geometry to generate electrical sensing signal, and the other is self-calibration feature that uses the sensing signals in surrounding region to calibrate and localize the sensing signal of the touch point. In addition to the above 2 key features, the flexoelectret touch sensor exhibits high sensitivity (the highest sensitivity up to ~835 mV/N) with high linearity (*R*^2^ > 0.995), as well as high robustness of withstanding continuous test of 80,000 s and more than 8,000 mechanical cycles, and anti-interference of keeping insensitive to environmental noises, such as temperature fluctuations, light illumination, and humidity interference. This study shows a promising design paradigm for flexible touch sensor based on flexoelectricity to reproduce the curved geometry of indentation and realize the self-powered and self-calibrated tactile imaging with high sensitivity, linearity, robustness, and anti-interference capacity.

## Results

### Design and mechanism of skin-inspired flexoelectret touch sensor

The skin-inspired flexoelectret touch sensor is designed to consist of an electron-injected polytetrafluoroethylene (PTFE) film sandwiched by 2 polydimethylsiloxane (PDMS) layers, providing a soft and transparent nature (Fig. 1B and Fig. S5). In reality, the real skin including hairy skin and glabrous skin has an average thickness of 1 to 3 mm [32], and its indentation depth (*d*) is approximately linear with the depressed force (*F*) [33], as observed on the forearm skins of 6 participants (blue points and line, Fig. 1C). To reasonably mimic the mechanical properties of real skin, the thickness of flexoelectret touch sensor is set to 2 mm, and its *d*-*F* characteristics (red line, Fig. 1C) matches that of the real forearm skin, covering the *F* range from a gentle finger press (<100 gram-force) to a relatively heavy press (100 to 500 gram-force) in real life.

Figure 1D shows the transduction mechanism of skin-inspired flexoelectret touch sensor. Before touch, the flexoelectret touch sensor has a mirror-symmetric structure and generates a symmetric distribution of electric field (*E*_s_) due to the injected electrons, resulting in no potential difference between the upper surface and the lower surface (top, Fig. 1D). During touch, the depressed device presents a curved geometry and produces a nonsymmetric electric field (*E*_non-s_), leading to a potential difference Δ*V* between 2 surfaces (middle, Fig. 1D). Δ*V* rises with *d* as the curved geometry-induced nonsymmetry increases. Furthermore, as the touch point moves, the curved region also moves, resulting in a new potential distribution at the new position (bottom, Fig. 1D). By analyzing the potential distribution, both the strength and position of the touch can be characterized.

To clarify the above transduction mechanism, we applied periodic indentation (black) on the flexoelectret touch sensor and simultaneously measured the output current with (red) and without (blue) injected electrons over time (Fig. 1E). Indentation is analogous to the effect of a finger or other object pressing into the device. The output current of the device with injected electrons is 3 orders of magnitude larger than that without injected electrons, indicating that the output current indeed originates from the curved geometry-induced nonsymmetric electric field. Additionally, there is a phase shift of 90° between the indentation depth and the current. This suggests that the output electrical current is spontaneously generated due to the potential difference from the curved geometry-induced nonsymmetric electric field: If the current is 90° out of phase with the indentation depth, the transported charge Δ*Q* (the induced charge difference) between the upper electrode and the lower electrode (from which the current is the time derivative) is in phase with the indentation depth.

There are 2 points worth noting: one is that the output electrical response directly related to the indentation is not an intrinsic flexoelectric response from nonsymmetric polarization induced by strain gradient in distorted lattice, but a kind of flexoelectric-like response from nonsymmetric electric field (distribution) induced by the curved geometry of device; the other is that the output electrical response is induced by the indentation (touch) and there is no external power source involved, indicating that the flexoelectret touch sensor possesses self-powered sensing capability.

### Simulations verifying the mechanism of self-powered transduction

**Fig. 2.**
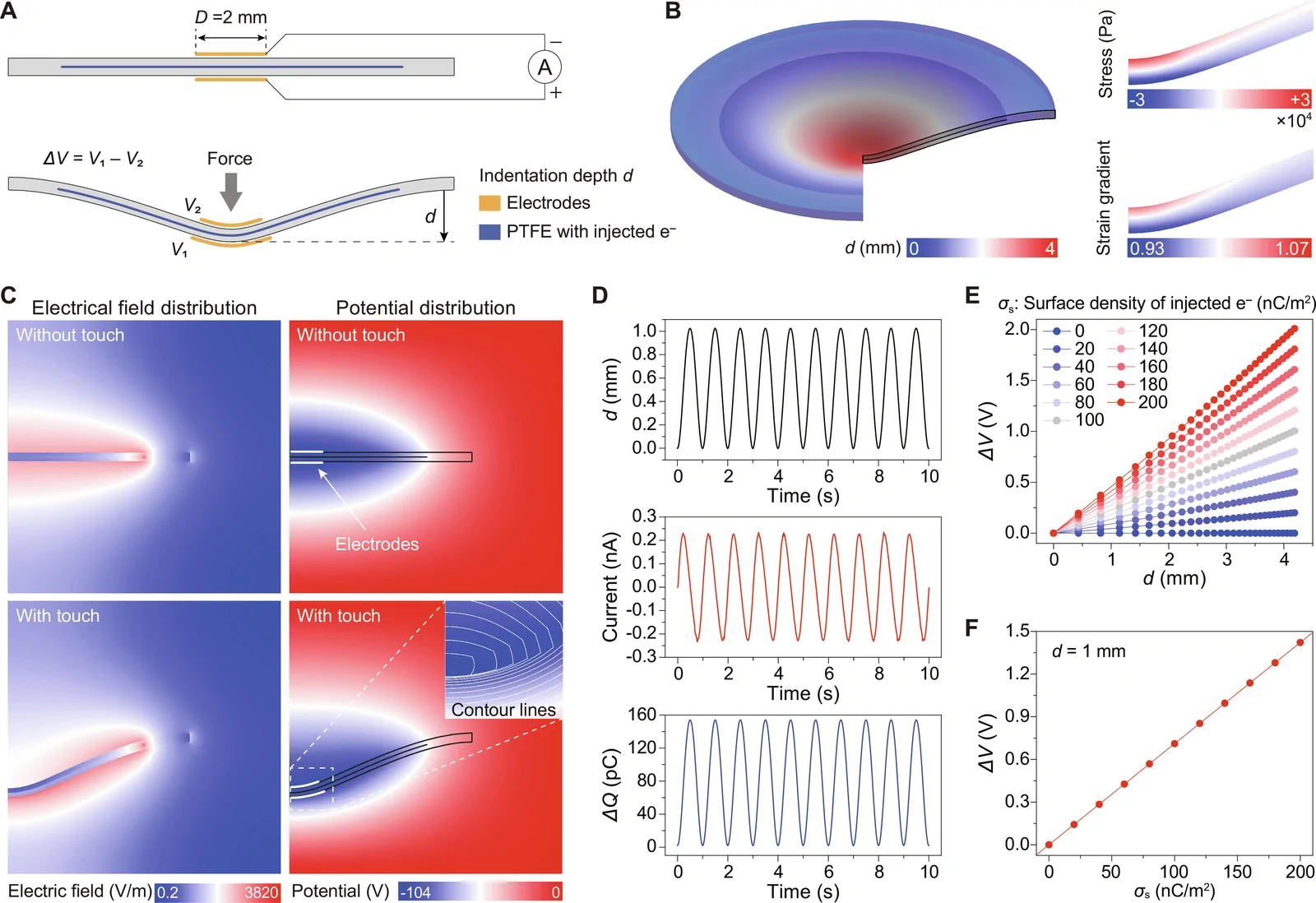
Simulations verifying the self-powered transduction mechanism of the flexoelectret touch sensor. (A) Schematic of the FEA simulation model for the flexoelectret touch sensor. (B) Indentation depth (*d* ~ 4.0 mm) along with distributions of stress and strain gradient under touch. (C) Distributions of electric field (left) and potential (right) with and without touch. (D) Waveforms of indentation depth, indentation-generated current, and the corresponding transported charge Δ*Q*. (E) Potential difference Δ*V* between upper and lower electrodes as a function of indentation depth (*d*) for the flexoelectret touch sensor with different surface densities (*σ*_s_) of injected electrons. (F) Linear relationship between Δ*V* and *σ*_s_.

We conducted finite element analysis (FEA) to verify the self-powered transduction mechanism. To demonstrate the concept of flexoelectret touch sensor, a FEA model with the detailed structure parameters of a single element is exhibited in Fig. 2A. The simulated indentation depth *d* is ~4.0 mm at the center point of the model’s upper surface under touch, and the distributions of stress and strain gradient are shown in Fig. 2B, indicating that the upper and lower surfaces experience nonsymmetric deformations. Additionally, a curved geometry of the flexoelectret touch sensor with a relatively constant radius of curvature (~1 m) is obtained across the device thickness on the touch point.

Furthermore, the distributions of electric field (left) and potential (right) simulated in Fig. 2C reveal a nonsymmetric electric field and a potential difference Δ*V* between 2 surfaces, resulting from the touch-induced curved geometry of device. The output current *I* and transported charge Δ*Q* simultaneously with the change of the indentation depth (*d*) over time are presented in Fig. 2D. It can be observed that the current response has a clear 90° phase shift, whereas Δ*Q* is in phase with *d*, as implied by the experimental result in Fig. 1E.

Excitingly, a linear dependency of the voltage response Δ*V* on the indentation depth *d* is found in Fig. 2E for the device with different surface densities (*σ*_s_) of injected electrons. This linear relationship between Δ*V* and *d* in the flexoelectret touch sensor conforms to the flexoelectric-like effect (Fig. S7). Figure 2F also shows a strong linear correlation between Δ*V* and *σ*_s_, suggesting that the nonsymmetric electric field generated by injected electrons is indeed responsible for the output electrical response as expected.

### Self-powered sensing performance of flexoelectret touch sensor

To evaluate the self-powered sensing performance of the flexoelectret touch sensor, we periodically applied the mechanical stimuli in forms of depressed force and indentation on the device and simultaneously measured the output voltage response Δ*V*.

**Fig. 3.**
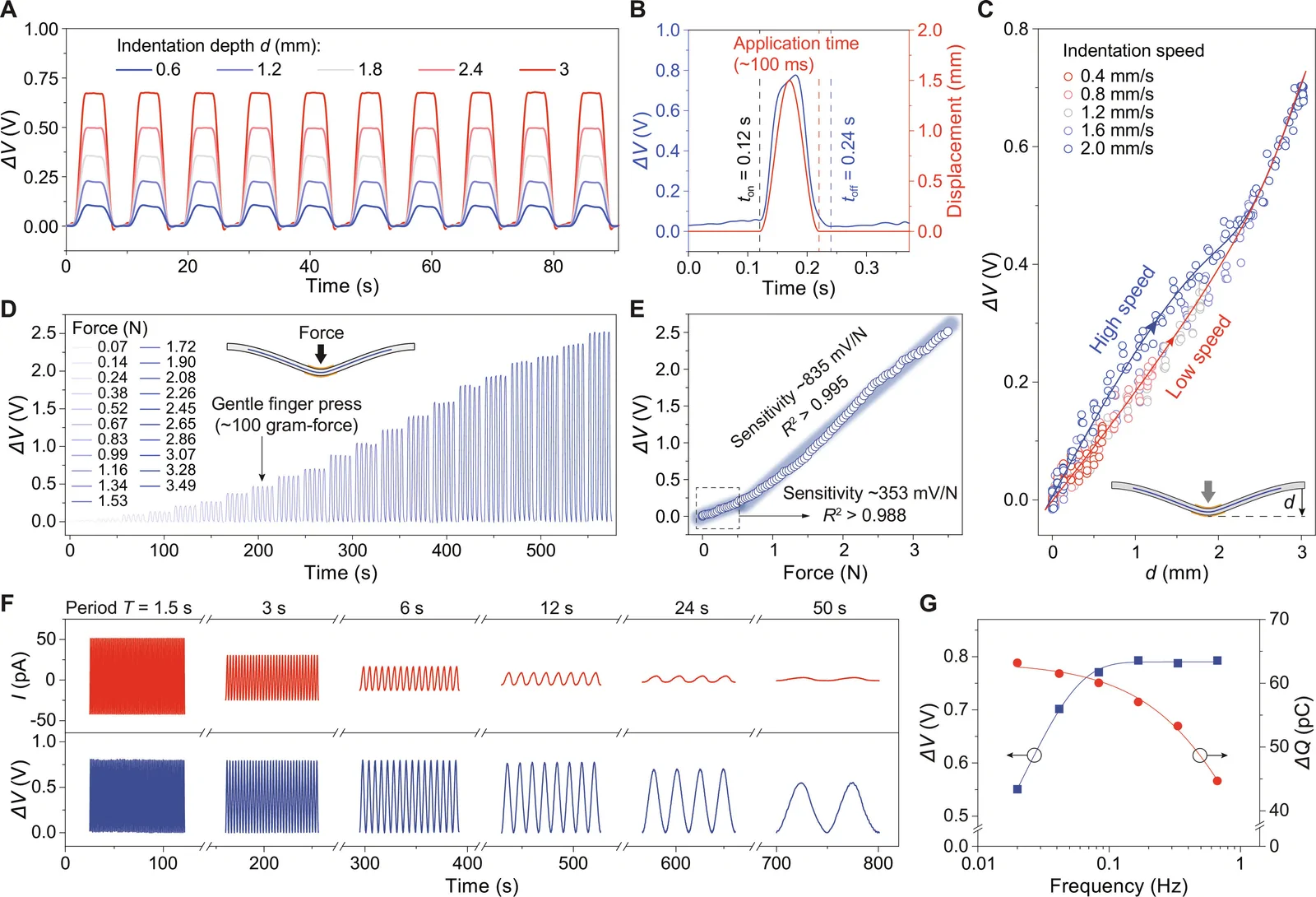
Self-powered sensing performance of the flexoelectret touch sensor. (A) Indentation-generated Δ*V* of the flexoelectret touch sensor in real time under actions of 10 on/off cycles with the indentation depth *d* ranging from 0.6 to 3.0 mm. (B) Response time of the flexoelectret touch sensor (*τ <* ~20 ms). *t*_on_ and *t*_off_ are respectively the rising time and falling time of the voltage response Δ*V*; the loading time of the external stimulus (application time) is ~100 ms. (C) Feature Δ*V*–*d* curves with various indentation speeds ranging from 0.4 to 2.0 mm/s. (D) Force-generated Δ*V* of the flexoelectret touch sensor in real time under actions of 5 on/off cycles with the applied force ranging from 0.07 to 3.49 N. (E) Δ*V* as a function of force ranging from 0.00 to 3.50 N. (F) Output current *I* and voltage Δ*V* of the flexoelectret touch sensor under force at different frequencies. (G) Δ*V* and Δ*Q* as functions of the frequency of applied force.

Figure 3A shows the highly reversible and stable Δ*V* over time under actions of 10 loading/unloading cycles with different indentation depths *d* ranged from 0.6 to 3.0 mm. The corresponding baseline without indentation remains relatively stable throughout the test; Δ*V* changes immediately upon applying *d*, and the amplitude of the response monotonically increases with increasing *d*. The response time of the flexoelectret touch sensor is supplied in Fig. 3B. The applying time of the external stimulus could be controlled by the linear motor in ~100 ms, and the corresponding peak width of the voltage response is about 120 ms. Therefore, we evaluate the response time of the flexoelectret touch sensor less than ~20 ms.

Further analysis on the dynamic voltage response shows that the Δ*V*–*d* characteristic curve is slightly related to the indentation speed (Fig. 3C). As the speed is less than or equal to 1.6 mm/s, the curves for different speeds are highly overlapped, and their average fitted curve (red line) presents linear at small *d* (0.0 to 2.0 mm) and slightly upturned at larger *d* (>2.0 mm). However, when the speed reaches 2.0 mm/s, the curve (blue line) shifts upward slightly at small *d*. This slight shift may result from the excessive compressive deformation of upper PDMS layer due to the instantly high speed (2.0 mm/s), which presumably increases the potential difference between the upper and lower surface of device. By calculating the slope of characteristic curve, we obtained a low-speed sensitivity of 0.181 mV/mm and a high-speed sensitivity of 0.238 mV/mm. Thus, the results indicate that the flexoelectret touch sensor is sensitive to transient mechanical signals, which are transmitted efficiently in the real skin.

As can be seen in Fig. 3D, during the application of loading/unloading cycles with different depressed forces *F* ranging from 0.07 to 3.49 N, the voltage response Δ*V* exhibits good stability and changes significantly with *F*, while the baseline remains around 0 V. A gentle finger force (~100 gram-force) can generate a Δ*V* of about 470 mV, which reaches the common target that should be accurately detected by on-skin electronics [7,8]. Furthermore, another characteristics of real skin (including hairy skin and glabrous skin), the dynamic response to *F*, is typically correlated linearly with the indentation depth as well as the level of curved geometry of skin [33]. In experiment, the flexoelectret touch sensor possesses 2 linear intervals: At external force <0.5 N, the sensitivity of the flexoelectret touch sensor is ~353 mV/N (*R*^2^ > 0.988), while at pressures ranging from 0.5 to 3.5 N, the flexoelectret touch sensor has an excellent sensitivity up to ~835 mV/N with high linearity (*R*^2^ > 0.995). It has proved that a limited detectable force is as small as approximately 1.2 mN, which is comparable to the magnitude of a gentle finger touch (on the order of 1 mN). The linear transduction mechanism is determined by the intrinsic relationship between the curved geometry-induced indentation (*d*) and the linear variation of potential difference (Δ*V*) (Fig. 2 and Fig. S7), overcoming the nonlinear response that is typical of many other sensitive transduction mechanisms (e.g., force-dependent capacitance distance in some capacitive types [34], force-dependent conductive path or magnitude in some piezoresistive types [35] and piezotronic types [11], and some other types [16,36]). Note that the above properties are obtained from the device with thickness of 2.0 mm to imitate real skin. Actually, higher sensitivity could be obtained to adapt to various application scenarios by reducing the thickness of the PDMS layer or increasing the surface density of injected electrons (Fig. 2E and F and Figs. S7 and S9).

The frequency-dependent sensing performance of the flexoelectret touch sensor is also tested and illustrated in Fig. 3F and G. As the frequency of depressed force decreases, the amplitude of output current *I* is dramatically reduced. Notably, the output voltage Δ*V* primarily drops below 0.1 Hz but remains relatively constant above this frequency. This indicates that the signal of Δ*V* is suitable for touch sensing with frequency above 0.1 Hz, which is sufficient to meet the demand for applications like finger pressing. By integrating the current from Fig. 3F to obtain the transferred charge Δ*Q* in one cycle, we found that Δ*Q* rapidly drops above 0.1 Hz. As an alternative strategy, the signal of Δ*Q* might be more appropriate for sensing touch with frequency below 0.1 Hz. These 2 sensing signals can perfectly compensate for the lack of their respective detection bandwidths.

### Robustness and anti-interference capacity of flexoelectret touch sensor

**Fig. 4.**
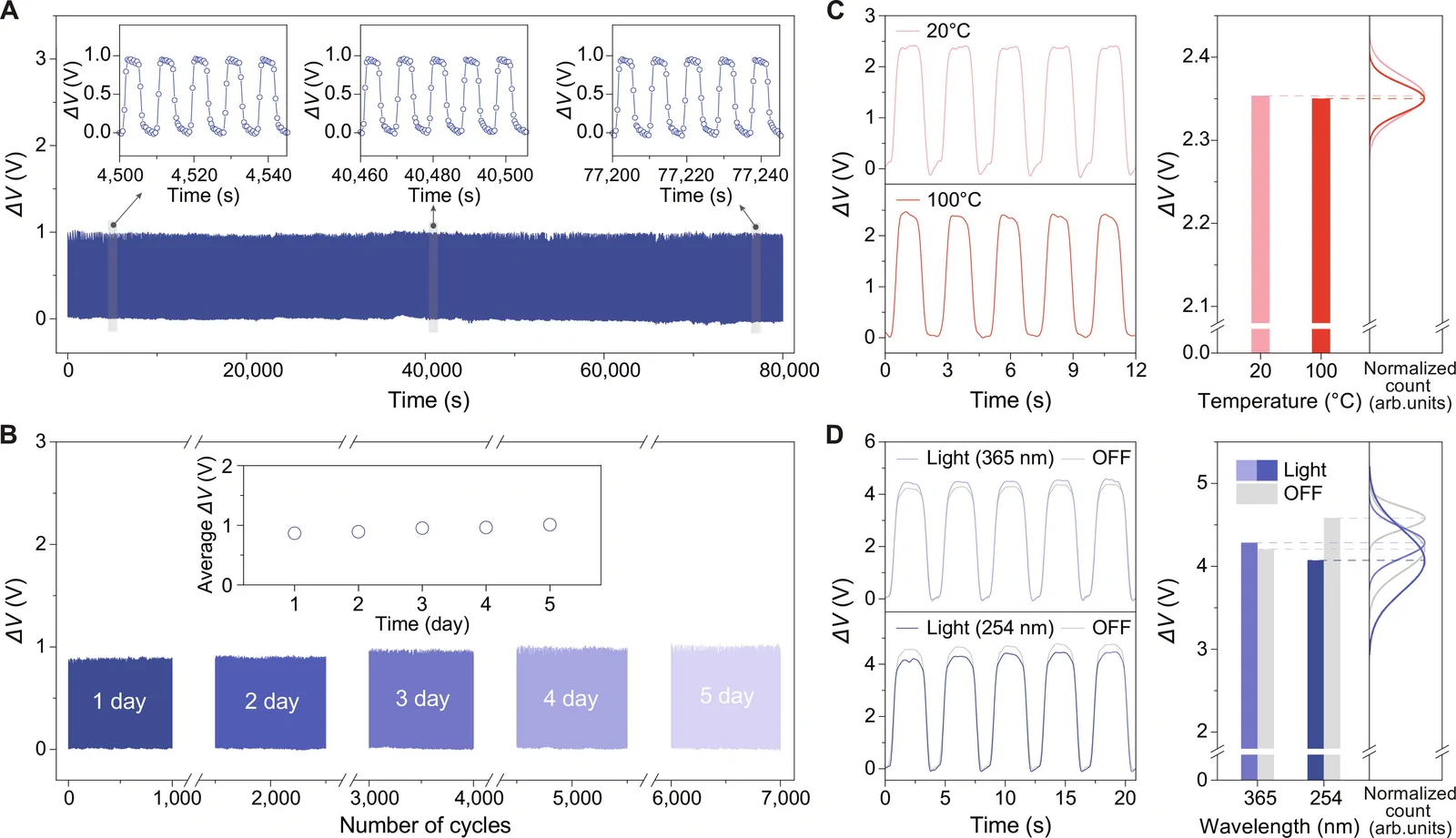
Robustness and anti-interference capacity of the flexoelectret touch sensor. (A and B) Long-term stability of the flexoelectret touch sensor to withstand continuous cycle test of 80,000 s (A) and more than 8,000 cycles (B). The insets of (A) show details of 5 cycles at different time. The inset of (B) shows the daily average Δ*V*. (C and D) Δ*V* of the flexoelectret touch sensor and the average value at 20 and 100 °C (C), and under illumination with wavelengths of 254 and 365 nm (D).

Robustness is a factor that must be considered for the application of touch sensors. Experimentally, as shown in Fig. 4A, the flexoelectret touch sensor is able to withstand continuous cycle test of 80,000 s without any obvious degradation in sensing performance. Even after sustained usage (more than ∼8,000 cycles), the voltage signal still remains stable (Fig. 4B). This indicates the remarkable robustness of the flexoelectret touch sensor to keep high performance, which exhibits good reversibility and repeatability throughout the test. As a whole, the flexoelectret touch sensor has not only high sensitivity and linearity but also high robustness.

Furthermore, another important factor to consider for touch sensors is the ability to keep insensitive to environmental noises, such as temperature fluctuation or light illumination. This is particularly crucial for touch sensors based on semiconductors or principles of piezoelectric, piezoresistive, piezotronic, or piezoionic effects. These types of sensors are susceptible to changes in material properties caused by environmental noises, which will degrade the touch sensing performance. So, we should study if the flexoelectret touch sensor is influenced by environmental factors. As shown in Fig. 4C, the flexoelectret touch sensor remains unaffected by temperature fluctuation (20 to 100 °C). Moreover, Fig. 4D demonstrates that it also maintains its sensing performance under different ultraviolet light illumination (254 and 365 nm). More experimental results (including humidity stability, washability, and repeated bending test) reveal the excellent anti-interference capacity of the flexoelectret touch sensor in the complex environment (Figs. S12 to S14). By now, it can be concluded that the flexoelectret touch sensor not only exhibits good functionality under adverse conditions but also retains stable performance throughout all these experiments, demonstrating the characteristics of mechanical robustness and anti-interference capacity with highly sensitive and linear touch sensing.

### Self-powered and self-calibrated tactile imaging of flexoelectret touch sensor array

**Fig. 5.**
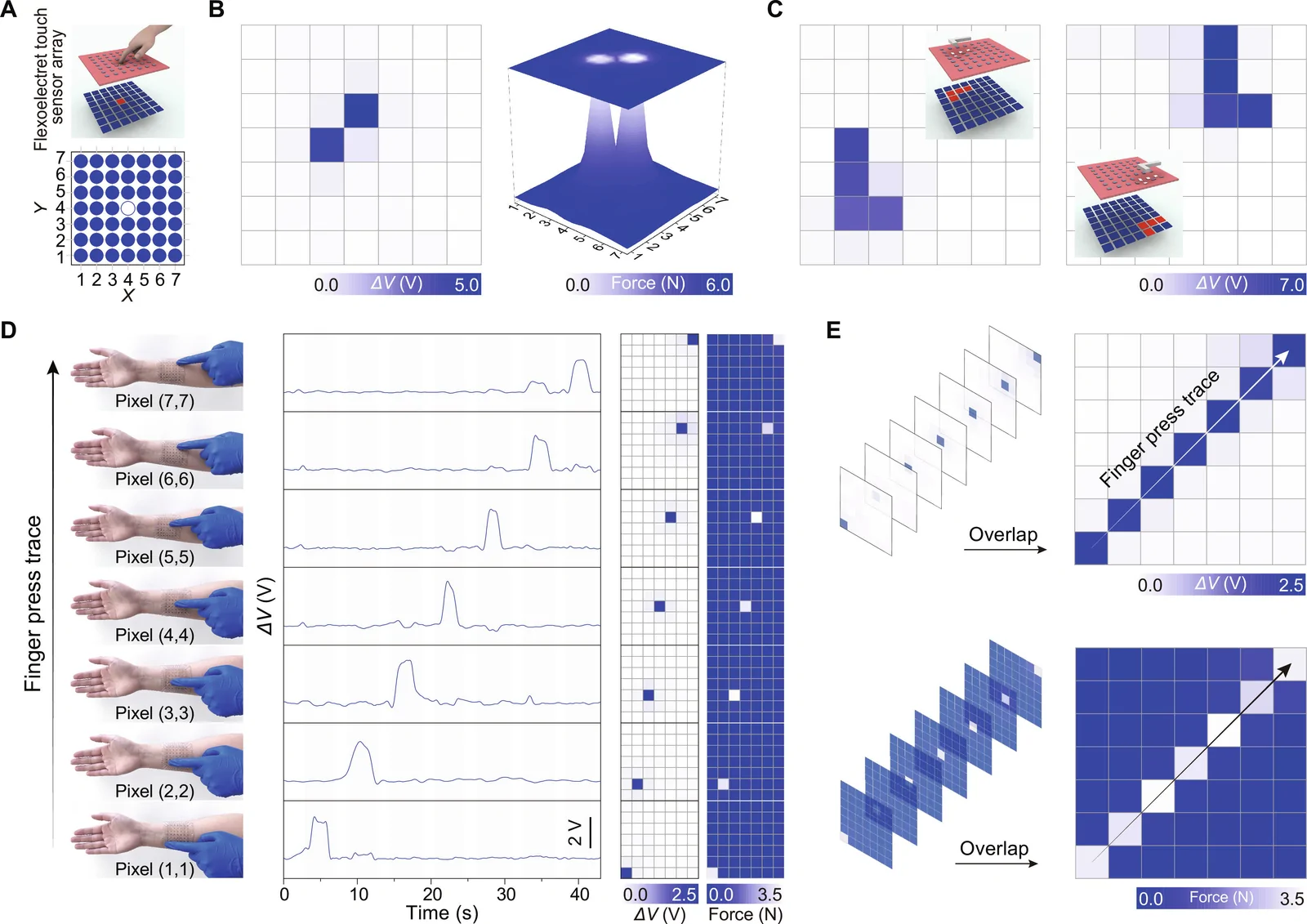
Self-powered flexoelectret touch sensor array with 7 × 7 pixels for tactile imaging, including shape-adaptive imaging and touch-trajectory imaging. (A) Schematic of the 7 × 7-pixel flexoelectret touch sensor array. (B) Δ*V* mapping and corresponding tactile imaging of 2-finger press. (C) Shape-adaptive imaging of an “L”-shaped object pressing different regions of the flexoelectret touch sensor array. (D) Photographs of the flexoelectret touch sensor array on a wrist (left), Δ*V* of pixels over time (middle), and dynamic Δ*V* mappings and corresponding force distributions (right) of a finger sequentially pressing pixels along a path: (1,1) → (2,2) → (3,3) → (4,4) → (5,5) → (6,6) → (7,7). (E) Touch-trajectory imaging obtained by overlapping Δ*V* mappings and force distributions at different time from (D).

To assess the performance of flexoelectret touch sensor in near real circumstance, a 7 × 7-pixel flexoelectret touch sensor array is fabricated for self-powered tactile imaging, including shape-adaptive imaging and dynamic touch-trajectory imaging in Fig. 5. The schematic of an array device prototype for self-powered tactile imaging is illustrated in Fig. 5A. When the spatial touch profile is applied on the array device by finger, the output voltage Δ*V* and the corresponding force distribution could be acquired by a multichannel measurement system. When 2 fingers touch the array device simultaneously, one with a slightly larger force [pixel (4,5)] and the other with a slightly smaller force [pixel (3,4)], the Δ*V* mapping (left, Fig. 5B) and the corresponding force distribution plot (right, Fig. 5B) are intuitively obtained. It is evident that the flexoelectret touch sensor array can accurately differentiate the adjacent touch points [pixel (4,5) and pixel (3,4)] and identify their respective strength. Moreover, the flexoelectret touch sensor array can also identify the position and the shape of an object pressed into it. By pressing an “L”-shaped object at different positions (the bottom-left corner and the top-right corner) of the device, the object’s position, shape, and depressed force can be reconstructed through 2-dimensional Δ*V* mapping (Fig. 5C). Since the flexoelectret touch sensor is sensitive to the touch-induced curved geometry, stronger response would be observed at the edges of object compared to the interiors, which is a special advantage of the flexoelectric-like transduction mechanism in the contour recognition of specific shape. The flexoelectret touch sensor array demonstrates the capacity for self-powered shape-adaptive imaging.

In addition to the above static imaging demonstrations, the flexoelectret touch sensor array can also implement the dynamic tracking for touch-trajectory imaging. When a finger touched different pixels along a specific path in sequence: (1,1) → (2,2) → (3,3) → (4,4) → (5,5) → (6,6) → (7,7) (left, Fig. 5D), the electrical signals Δ*V* with time of each channel in parallel are recorded continuously (middle, Fig. 5D). Then, through the calibration based on the voltage–force curve, we can obtain a series of force distributions over time from the Δ*V* mappings (right, Fig. 5D). Furthermore, by overlapping the Δ*V* and force distribution maps in chronological order, the real-time visualization for touch trajectory of the moving finger could be subsequently depicted (Fig. 5E), which demonstrates the capacity of flexoelectret touch sensor array for self-powered touch-trajectory imaging.

**Fig. 6.**
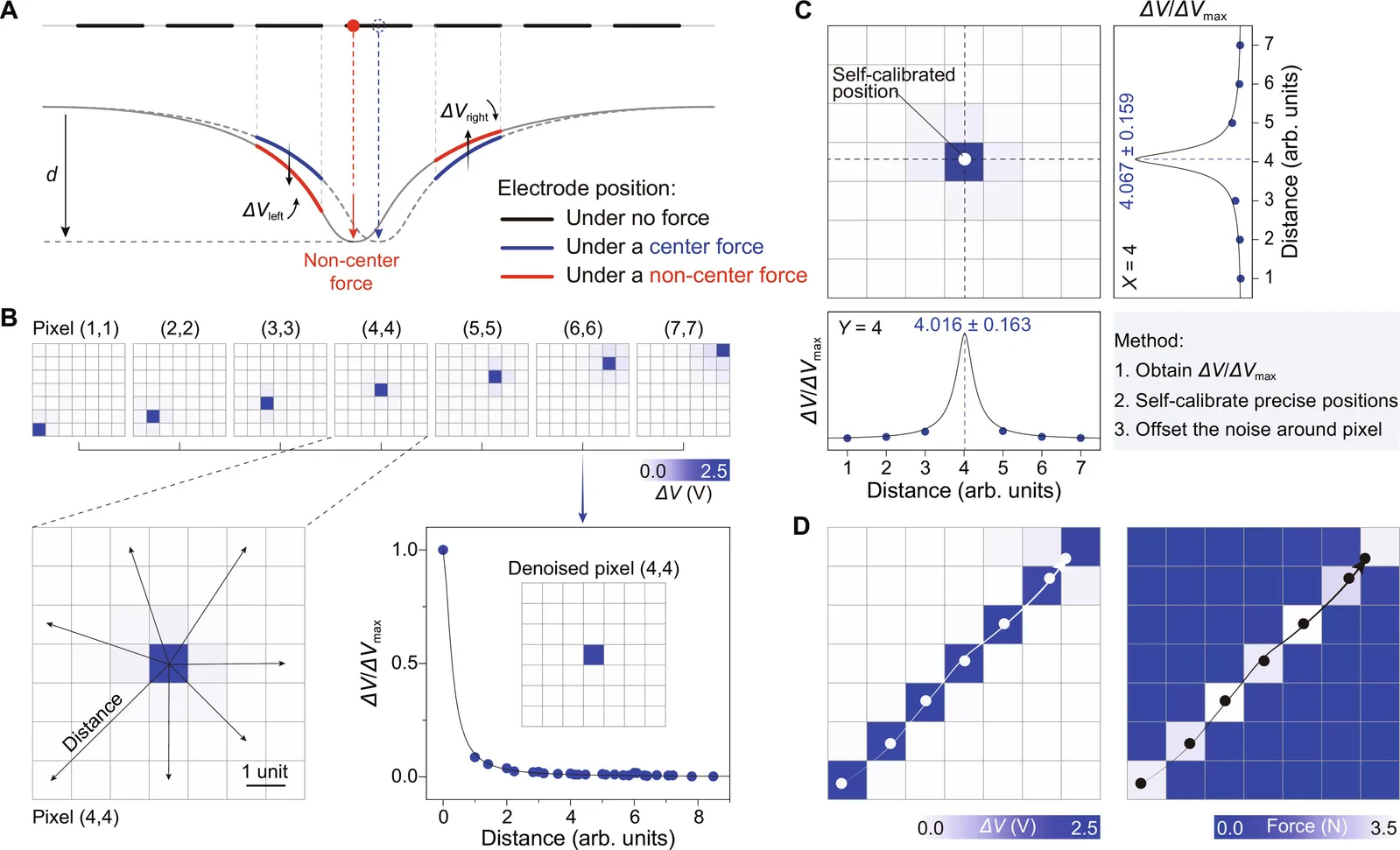
Self-calibrated flexoelectret touch sensor array for touch-trajectory imaging. (A) Self-calibration characteristics of the flexoelectret touch sensor array. Δ*V*_left_ and Δ*V*_right_ represent output Δ*V* of the left adjacent pixel (electrode) and the right adjacent pixel (electrode), respectively. (B) Normalized Δ*V* (Δ*V*/Δ*V*_max_) as a function of distance between pixels, learning from the strain interference between pixels of touch-trajectory imaging in Fig. 5D. Δ*V*_max_ is the Δ*V* of touch point. (C) Self-calibrated position of each touch point beyond the limit of pixel size by utilizing the function between Δ*V*/Δ*V*_max_ and distance obtained in (B). (D) Self-calibrated touch-trajectory imaging.

Different from conventional touch sensing mechanism based on piezoelectric, capacitive, piezoresistive, and other effects, the flexoelectret touch sensor array typically utilizes the touch-induced curved geometry to sense the touch, involving surrounding regions centered on the touch point. When a pixel is pressed down, the neighboring pixels around the point of application will generate response signals, which can be attributed to the mechanical strain interference spreading through the PDMS layer, rather than the electrical crosstalk. These noises are commonly detrimental to tactile imaging on the basis of conventional transduction mechanisms. However, due to the distinct mechanism, the strain interference of the neighboring pixels is determined by the curved geometry of device. As a result, we can utilize the associated information in neighboring strain interference signals to calibrate the position of touch point. As illustrated in Fig. 6A, when there is no touch, the profiles of electrodes evenly distributed on the flat surface. When a pixel is subjected to a center touch force (applied on the center of electrode, colored in blue), the 2 adjacent pixels would generate response signals (Δ*V*_left,0_ for the left adjacent pixel, and Δ*V*_right,0_ for the right adjacent pixel) with the same magnitude (Δ*V*_left,0_ = Δ*V*_right,0_). When a pixel is subjected to a noncenter touch force (applied on the left edge of electrode for example, colored in red), the left adjacent pixel would be closer to the nonsymmetric electric field induced by the curved geometry, leading to a larger response signal Δ*V*_left_ (> Δ*V*_left,0_). In contrast, the right adjacent pixel would be further away from the nonsymmetric electric field, leading to a smaller response signal Δ*V*_right_ (< Δ*V*_right,0_). Therefore, according to the distribution of strain interference signals in neighboring pixels, we can localize the exact force point of the touch pixel. In other words, the imaging quality could be further improved by analyzing the measured signal itself to self-calibrate.

Learning from the experimental data of touch-trajectory imaging in Fig. 5D, we can obtain the relationship between the normalized Δ*V* (the output voltage is normalized by the maximum voltage of the touch pixel, Δ*V*/Δ*V*_max_) of pixels and their distance from the touch pixel (Fig. 6B). Utilizing this statistical relationship (reflecting the strain interference between neighboring pixels), we could more accurately localize the touch point beyond the pixel size limit (Figs. S15 and S16). For instance, as shown in Fig. 6C, based on the strain interference distribution around pixel (4,4) (the bottom for *x* axis and the right for *y* axis), the actual touch position of the finger can be identified as the white point inside the pixel (4,4). The error of the touch position is about 0.16 unit of pixel size (7.0 mm), equal to 1.12 mm, which is close to the size of the receptive fields of mechanosensory neurons in human fingertip (1 to 2 mm diameter) and smaller than that in palm (5 to 10 mm diameter) and still smaller in parts like arm [30]. Consequently, the touch-trajectory imaging in Fig. 5E can be optimized by the self-calibration process, and a more precise tactile imaging is achieved in Fig. 6D. Furthermore, it is anticipated that by combining the use of multi-pixel sensor as artificial neural network strategy [37], the flexoelectret touch sensor array holds great potential for higher resolution and expanded capabilities.

## Discussion

In summary, a flexoelectret touch sensor mimicking the depressed skin with curved geometry has been proposed and developed, which can effectively utilize the touch-induced curved geometry to realize self-powered and self-calibrated tactile imaging. In addition, it also exhibits high sensitivity (the highest sensitivity up to ~835 mV/N) with good linearity (*R*^2^ > 0.995), as well as excellent robustness for withstanding continuous cycle test of 80,000 s and over 8,000 mechanical cycles, and anti-interference for keeping insensitive to temperature fluctuations, light illumination, and humidity interference. Demonstrations of the flexoelectret touch sensor array for tactile imaging, including shape-adaptive imaging and touch-trajectory imaging, reveal the wide-ranging applicability of this on-skin technology to promote the advances in next-generation human–machine interfaces, behavioral monitoring, healthcare, and robotics.

## Materials and Methods

### Fabrication of the flexoelectret touch sensor

The flexoelectret touch sensor is composed of a PTFE thin film as the charged central layer, which is sandwiched between 2 PDMS substrates and fused together. The PDMS substrates are prepared by mixing a prepolymer and a curing agent of the commercially available silicone elastomer Sylgard 184 (Dow Corning) in a weight ratio of 10:1. The resulting mixture of PDMS is then transferred into the mold, followed by 10 min of degassing in vacuum. After curing at 60 °C for 4 h, the solidified PDMS layers with thickness of approximately 1 mm can be obtained when peeled off from the mold (Fig. S5A).

In the next step, the corona charging treatment is performed to inject electrical charges into the PTFE thin film. The 30-μm-thick PTFE thin film is cut into the desired shape with dimension of 50 mm × 50 mm before being cleaned with ethanol and deionized water. In the charging process, the intense electric field of the corona electrode (the array of needle tips) can generate a high quantity of charge carriers from the ionized gas [38,39]. Acceleration by the electric field of grid electrode, the injected charges would be uniformly distributed over the surface of PTFE thin film. As shown in Fig. S5B, the PTFE thin film is laid on a metal plate in the distance of approximately 10 mm from the needle tips, while the grid electrode is placed between them. During charging, the charging voltages applied on the corona electrode and the grid electrode are –15 kV and –9 kV, respectively, using 2 DC high-voltage power sources (HB-Z203-1AC, Tianjin Hengbo).

To fabricate the flexoelectret touch sensor prototype, a PTFE thin film is charged (Fig. S6) and then transferred to the as-prepared PDMS substrate covered with some PDMS mixture as adhesion. A second PDMS substrate with PDMS mixture coated on its underside is carefully attached on top of the PTFE thin film to encapsulate the structure. After degassing, the sample is put in an oven (DHG-9030A, Shanghai Yiheng) and both PDMS substrates and the PTFE thin film are thermally (100 °C for 1 h) bonded together as a whole. The resulting thickness of the device is approximately 2 mm, since the contribution from the thin film can be ignored. Later, the patterned metal layers (Pt/Cr; Pt: ~100 nm and Cr: ~10 nm) as the top/bottom electrodes are deposited using an RF (radio frequency) magnetron sputtering (RF power: 150 W, Ar gas flow: 50 sccm (standard cubic centimeter per minute), and working pressure: 0.5 Pa) onto both sides of the substrate through the mask (Fig. S5C). Finally, the copper (Cu) wires are attached on each electrode using silver (Ag) paste to provide good electrical connection between the flexoelectret touch sensor and the external measurement circuit.

### Characterizations and measurements

To characterize the sensing performance of the flexoelectret touch sensor, the device is connected to the specific electrical measurement system (Fig. S17). The current generated by the flexoelectret touch sensor is detected by a low-noise current preamplifier (SR570, Stanford Research Systems), which is acquired by a data acquisition card (PCI-6259, National Instruments) and then analyzed by a computer using LabVIEW program simultaneously during testing (Fig. S17A). Particularly, the potential difference of the flexoelectret touch sensor is measured using a low-noise preamplifier (SR560, Stanford Research Systems) and a digital source meter (2636B, Keithley).

In the experiments for cycle testing, a displacement-controlled linear motor (E1100, LinMot) is programmed to apply the periodical force on the flexoelectret touch sensor, using a loading pillar with spherical indenter (diameter of *D* ~ 5.0 mm). Unless otherwise stated, the device is fixed to an 80-mm-diameter acrylic sample holder with cylinder-shaped supporting edge (Fig. S17B).

To evaluate the anti-interference performance, the voltage response of the device for the thermal stability is recorded in the oven, by changing temperature from 20 to 100 °C. Subsequently, the device is perpendicularly illuminated on the surface at room temperature using a portable ultraviolet lamp (ENF-280C, Spectroline).

For tactile imaging, the contact object is adjusted and aligned by a home-made mechanical stage, which is composed of a 3-axis linear motion system (FSK40XYZ-L, Chengdu Fuyu) and a digital force gauge (DS2, Zhiqu) mounted vertically.

## Data Availability

The datasets used and analyzed during the current study are available from the corresponding authors on reasonable request.

## Supplementary Materials

Supplementary 1 Figs. S1 to S17 Table S1 Notes S1 to S11 References [40–74]
