# Supplementary material for: Skin-Inspired Flexoelectret Touch Sensor for Self-Powered and Self-Calibrated Tactile Imaging
Source: Research (Wash D C). 2026 Aug 31;9:1419. doi: 10.34133/research.1419 (PMC13527212; doi:10.34133/research.1419)
Supplement: Supplementary file 1 [file research.1419.f1.docx]

**Supplementary Information for**

**Skin-inspired Flexoelectret Touch Sensor for Self-powered and Self-calibrated Tactile Imaging**

Rui Ge^1,2^†, Qiuhong Yu^2,3^†, Yongkang Zhang^2^†, Yafeng Cai^2^, Shuhai Liu^2*^, Weimin Liu^5^, and Jun Zhang^4^, Yong Qin^2,4*^

^1^College of Information and Communication Engineering/College of integrated circuits, Harbin Engineering University, Harbin, Heilongjiang 150001, China.

^2^Institute of Nanoscience and Nanotechnology, School of Materials and Energy, Lanzhou University, Lanzhou, Gansu 730000, China.

^3^Henan Key Laboratory of Photoelectric Energy Storage Materials and Applications, School of Physics and Engineering, Henan University of Science and Technology, Luoyang, Henan 471000, China.

^4^State Key Laboratory of Environment Characteristics and Effects for Near-space, School of Interdisciplinary Sciences, Beijing Institute of Technology, Beijing 100081, China

^5^State Key Laboratory of Solid Lubrication, Lanzhou Institute of Chemical Physics, Chinese Academy of Sciences, Lanzhou 730000, China.

†These authors contributed equally to this work.

^*^Address correspondence to: liushuhai1991@live.cn and qinyong@lzu.edu.cn

**This PDF file includes:**

**Notes S1-S11**

**Figures S1-S17**

**Table S1**

**Note S1. Flexoelectric-like effect in the flexoelectret**

**(1) Intrinsic flexoelectricity**

As we know, the flexoelectricity is an electromechanical property that enables the mechanical manipulation of polarization by the coupling effect between the local electrical polarization and the strain gradient in dielectrics. Such inhomogeneous strain field can break any materials’ inversion symmetry owing to the distorted crystal structure, resulting in a polarization along the strain-gradient direction [40]. Unlike piezoelectric effect, which occurs only in non-centrosymmetric materials, the flexoelectric effect induced by strain gradients is present in all symmetry classes of materials [24]. For a simple ionic lattice, uniform strain does not break inversion symmetry, and hence cannot generate polarization in a centrosymmetric material (**Fig. S1A**). As illustrated in **Fig. S1B**, a strain gradient, however, can break inversion symmetry, therefore leading to relative displacements of the centers of negative and positive charges and to the appearance of polarization in any material [23].

However, the charge density that can be generated by flexoelectricity is normally small, which limits its practical application [25]. The flexoelectric response of a material is directly proportional to its flexoelectric coefficient and the strain gradient, which can be represented mathematically by the following equation:

$$\begin{aligned} P_{l}=\mu_{\mathrm{ijkl}}\frac{\partial u_{\mathrm{ij}}}{\partial x_{k}}\#\text{(1)} \end{aligned}$$

where *P*_l_ is the induced flexoelectric polarization, ∂*u*_ij_/∂*x*_k_ is the strain gradient, and *μ*_ijkl_ is the flexoelectric coefficient [41]. Notably, strong flexoelectricity is expected in materials with high-dielectric constants. Following this way, relatively high flexoelectric coefficients are observed in certain ferroelectric materials, including lead magnesium niobate ceramics, barium strontium titanate, lead zirconate titanate ceramics, and barium titanate (BTO). Apart from the aforementioned rigid materials, polymers, and soft elastomers are naturally flexible and can undergo significant deformation and tolerate substantial strain gradients [42]. However, they exhibit much lower flexoelectric coefficients (≈10^−8^ to 10^−9^ C·m^−1^) which is several orders of magnitude smaller than that for ferroelectric materials [43]. Thus, it is necessary to further enhance the flexoelectric coefficient of polymers in order to achieve a strong flexoelectric effect [44].

**Fig. S1. Schematic illustration of the microscopic mechanism of flexoelectricity** **based on an ionic crystal [23].** (**A**) Zero net polarization under homogeneous deformation (e.g., uniaxial compression) due to the overlap of positive and negative charge centers. (**B**) When experienced an inhomogenous strain (e.g., bending), centers of positive and negative charge would mismatch thus creating a non-zero polarization (arrow) due to the strain gradient [45].

**(2) Flexoelectric-like effect**

To further enhance the flexoelectric performance of materials, a more effective way might be employing flexoelectric-like effects. The flexoelectric-like effect indicates a phenomenon which is not caused by the intrinsic flexoelectricity but enables the material to perform like those with high flexoelectricity. The flexoelectric-like effect may be created in all kinds of materials through different mechanisms [46]. For example, Narvaez *et al.* showed the large flexoelectric-like response in semiconductors enhanced by increasing conductivity, which could be explained by a barrier layer mechanism [25]. According to the generalized definition of the flexoelectric effect (generating electric polarization by a strain gradient), this flexoelectric-like response belongs to the flexoelectric effect [24].

**(3) Conventional electret-based materials**

An electret is a dielectric material that permanently holds charges or electric dipole moments. A well-known example for the application of electret materials is the piezoelectret (or ferroelectret). In piezoelectret materials (PP as an example), abundant air voids (from ~1 to ~100 μm) are distributed inside the electret architecture. After charging, several positive/negative electric charges would accumulate on the inner top/bottom surfaces of the air bubbles, forming macroscopic ‘quasi-dipoles’, as shown in **Fig. S2A**. Due to the ‘quasi-dipoles’, the electret-based the cellular film exhibited effective piezoelectric properties analogous to those of traditional piezoelectric materials (**Fig. S2B**) [38].

**Fig. S2. Schematic illustration of the piezoelectric effects of piezoelectrets [38].** (**A**) The piezoelectret structure after charging shows that the upper and lower surfaces of the voids are oppositely charged. (**B**) The piezoelectret exhibits effective piezoelectricity due to the movements of macroscopic ‘quasi-dipoles’ and electrostatic induction.

**(4) Simulations analysing the mechanisms of flexoelectret and piezoelectret**

Inspired by piezoelectret (or ferroelectret), Wen *et al.* fabricated a flexoelectret with tunable flexoelectric-like response through introducing space charges, where the flexoelectric coefficient in silicon rubbers was increased by more than two orders of magnitude [9].

The underlying physical mechanism is systematically clarified in **Fig. S3**. The structure of a flexoelectret is schematically shown in **Fig. S3A**, embedding a layer of charges on the middle plane of dielectric material (e.g., PDMS) [44]. Due to the deposited charges, the material is polarized and the induced polarizations above and below the charge layer have exactly the same magnitude *P*_0_ but opposite directions (**Fig. S3A-i**). Under uniform compression, the shape is still symmetric about the middle plane. As shown in **Fig. S3A-ii**, the polarizations of two parts still have the same magnitude *P*_1_ and opposite directions. Thus, the overall polarization of the flexoelectret is still zero, showing no effective piezoelectricity. While if subjected to a bending deformation, the upper and lower parts of the flexoelectret would experience totally different deformations, leading to strains opposite in sign (**Fig. S3A-iii**) [46]. The compressive and tensile strains induced by bending cause the magnitudes of polarization *P*_2_' and *P*_2_" are no longer the same, which generates net polarization along the thickness direction. The overall polarization result in a potential difference on the upper and lower surfaces and thus an effective flexoelectric-like response.

As a comparison, in the case of a piezoelectret with homogeneous piezoelectricity, the same piezoelectric coefficient is distributed along the thickness direction, as shown in **Fig. S3B-i**. When the piezoelectret undergoes uniform compression deformation (**Fig. S3B-ii**), a decrease in the thickness leads to a lower charge-separation distance of the ‘quasi-dipole’, resulting in a weaker polarization (*P*_1_ < *P*_0_) and a high macroscopic piezoelectric response (as discussed above). However, due to the opposite strains induced by bending in **Fig. S3B-iii**, the increased and decreased magnitudes of polarization (*P*_2_' > *P*_0_ > *P*_2_") above and below the neutral plane will almost offset each other, resulting in a charge cancellation effect and a small flexoelectric-like response [24, 46].

**Fig. S3. Schematic illustration of the transduction mechanisms of the flexoelectret and the piezoelectret.** Arrows represent polarization. The basic working modes of (**A**) the flexoelectret and (**B**) the piezoelectret in (i) initial state, and under (ii) uniform compression, or (iii) bending deformation.

In order to verify the mechanisms of the flexoelectret and piezoelectret, a series of finite element simulations (**Fig. S4**) are also conducted with inversion symmetry and mirror symmetry polarization distribution [24]. The schematic of the finite element model is shown in **Fig. S4A**, with a focus on analyzing two types of polarization distribution modes. The flexoelectret model is the inversion symmetric polarization (**Fig. S4A-i**), with a layer of charges (charge density, *σ*_s_ = -100 nC/m^2^) deposited on the middle plane. The piezoelectret model is the mirror symmetric polarization (**Fig. S4A-ii**), where the positive/negative polarization charges on the top/bottom surfaces are set to be *σ*_s_ = ±100 nC/m^2^. To investigate the electric potential responses of the flexoelectret and piezoelectret under bending deformation, various curvature radiuses (*r* ranged from 100 to 10000 mm) of the neutral (middle) layers are introduced in the models, corresponding to the curvatures ranging from 0.1 to 10 m^-1^ (**Fig. S4B**). Quantitative analysis indicates that the potential difference ∆*V* increases (from 0.0065 V to 0.61 V) almost linearly with respect to the curvature of flexoelectret model, indicating the flexoelectric-like response characteristics (**Fig. S4C-i**). In contrast, the magnitude of ∆*V* seems to have only slight changes (from 8.10 V to 8.07 V) for the piezoelectret model (**Fig. S4C-ii**), which is consistent with the previous analysis of the mechanisms (**Fig. S3B**).

**Fig. S4. Simulation analysis for the flexoelectret and the piezoelectret under bending deformation.** (**A**) Schematic depictions of the analytical models for (i) the flexoelectret with the negative electric charges (charge density, *σ*_s_ = -100 nC/m^2^) on the middle plane; and (ii) the piezoelectret with the positive/negative polarization charges (*σ*_s_ = ±100 nC/m^2^) on the top/bottom surfaces. (**B**) Simulation results of the potential distribution for (i) the flexoelectret and (ii) the piezoelectret under bending deformation. (**C**) Potential difference Δ*V* between the upper and lower electrodes as a function of curvature for (i) the flexoelectret and (ii) the piezoelectret.

**Note S2. Self-powered sensing effect**

“Self-powered” describes the intrinsic signal generation mechanism of the sensing device. [47] The self-powered sensing effects involve collecting energy from mechanical motion, light, and even chemical reactions without external energy to generate sensing signals. Under the circumstances, it acts as both an energy harvesting device and a sensing device. It’s worth emphasizing that the difference between the self-powered sensor and the self-powered sensing system. The former could achieve active zero-power sensing through the generation of sensing signal without consuming external energy, but still requires an external power source to capture and transmit sensing signals. The latter realizes the autonomous driving of the whole system by integrating energy harvesting, self-sensing, signal processing, and transmission modules efficiently. [48]

For the flexoelectret touch sensor, the mechanical indentation induces a curved geometry in the device, which in turn produces a non-symmetric electric field and a corresponding voltage output across the electrodes. The process of the signal generating is entirely driven by the mechanical stimuli of touch. No external power is supplied to the sensor to facilitate the electromechanical transduction. Consequently, the flexoelectret touch sensor can function as a passive, energy-harvesting device, which is in consistent with the first definition of “self-powered” sensing mentioned above. It is noted that while the sensor generates the voltage output signal via the flexoelectric-like effect, the experimental measurement relies on the external instruments for data acquisition. The present study does not claim to have achieved the second level of system-level autonomy.

**Fig. S5. Fabrication process of the flexoelectret touch sensor array.** (**A**) Schematic of preparation and assembly steps for the flexoelectret touch sensor device. (**B**) Experimental setup for corona charging. (**C**) Design of the patterned electrodes for the 7×7-pixel array structure.

**Note S3. Surface potential of the negatively charged PTFE film**

The charge storage property of the PTFE electret film could be characterized by surface potential measurement. According to the definition of surface potential:

$$\begin{aligned} V_{S}=\frac{d_{e}}{\varepsilon_{0}\varepsilon_{r}}\times\sigma\#\text{(2)} \end{aligned}$$

where *V*_S_ is the surface potential, *σ* is the surface charge density, *d*_e_ is the electret thickness, *ε*_0_ is the vacuum permittivity, and *ε*_r_ is the relative permittivity. Equation 2 indicates that *V*_S_ is proportional to *σ*, so surface potential is measured to reflect the real-time surface charge density of electret films. [49]

**Fig. S6. Surface potential of the negatively charged PTFE film.**

Before measurement, corona charging process is employed to inject charges onto electret film. are negatively (-15 kV) corona charged for 5 min, while the gate voltage (-9 kV) is utilized to enhance the charging effect. In the measurement of 2 weeks, the surface potential of PTFE keeps stable at about -3.1 kV (**Fig. S6**), exhibiting excellent property in storing negative charges. [44, 50]

**Note S4. Simulation analysis for the impact factors on optimizing output performance**

To further confirm the transduction mechanism of the flexoelectret touch sensor, deformation simulation analysis is performed using the software COMSOL Multiphysics. Considering that flexoelectricity is a linear coupling between strain gradient and polarization [51], it is necessary to investigate the electrical response of the flexoelectret touch sensor under the inhomogeneous deformation induced by curved geometry.

For convenience, we established 2D axisymmetric model based on the size and material parameters of the experimental specimen. As illustrated in **Fig. S7A**, the FEA model is formed by a 2-mm-thick PDMS substrate (*R*_PDMS_ = 40 mm) with a charge layer (*R*_PTFE_ = 30 mm) on the middle plane and electrodes (*R*_Electrode_ = 6 mm) on the lower and upper surfaces. Note that we introduced an assumption: the size of the neutral layer is unchanged when subjected to the curved deformation, which is usually characterized by curvature (*κ* = 1/*r*). Here, various curvature radiuses (*r* ranged from 100 to 2000 mm) of neutral layers are introduced in the FEA models, corresponding to the curvatures ranging from 0.5 to 10 m^-1^. Additionally, the main property parameters of the FEA model for the deformation simulation are taken from the material library database in COMSOL Multiphysics. For example, the relative permittivity, mass density, Young's modulus, Poisson's ratio of PDMS are set to be 2.75, 970 kg/m^3^, 750 kPa, and 0.49, respectively [52, 53].

**Figure S7B** and **S7C** demonstrate the FEA simulation results from the flexoelectret touch sensor with different surface densities (*σ*_s_) of injected electrons. It can be seen clearly that the voltage response ∆*V* increases almost linearly with respect to the sample curvature, indicating the flexoelectriclike response characteristics. As expected, the output voltage ∆*V* exhibits strong linear dependency on the charge density (*σ*_s_), which is consistent with the result in **Fig. 2F**.

In order to optimize the geometric structure of the flexoelectret touch sensor, we can use the output from the analytical model to guide device design. We systematically investigated the influences of electrode radius on the sensing performance in devices with the electrode structure ratio (defined as the radius ratio of the top/bottom electrode to the charge layer, *R*_Electrode_/*R*_PTFE_) varying from 0.05 to 1. The *R*_Electrode_/*R*_PTFE_ variations are made by changing the radius of top/bottom electrodes (*R*_Electrode_ ranged from 1.5 to 30 mm), keeping all other geometries the same. As demonstrated by the calculated curves of the voltage response ∆*V* as a function of curvature in **Fig. S7D**, the level of the voltage changing with the increased curvature is gradually enhanced as the electrode radius decreases. It is also noted that the voltage response ∆*V* increases approximately linear with the curvature when the electrode is relatively small, whereas the increasing trend of voltage becomes slower with the larger electrode. Under the application of largest curvature (*κ* = 10.0 m^–1^), the output voltage ∆*V* decreases significantly from 0.501 V at the electrode structure ratio of *R*_Electrode_/*R*_PTFE_ = 0.05 down to only 0.267 V at the ratio of *R*_Electrode_/*R*_PTFE_ = 1 (**Fig. S7E**). This result indicates that the sensing performance can be improved by reducing the size of electrode, suggesting that the flexoelectret touch sensor is especially adaptive for the miniaturization and integration of electronics.

Moreover, **Figure S7F** and **S7G** show the impact of varying the substrate thickness (*H*_PDMS_) on the output performance of the flexoelectret touch sensor, corresponding to the sample thickness ranging from 1.0 to 10.0 mm. Obviously, it is observed in **Fig. S7G** that the output voltage ∆*V* is directly proportional to the thickness, which follows the flexoelectriclike effect [44]. In addition, it is worth noting that the thickness of PDMS substrate will modify the mechanical property of the device, which means larger depressed force is needed to keep the same curvature. Therefore, the sensing performance of the flexoelectret touch sensor can be effectively controlled by selecting the appropriate thickness of substrate, while maintaining sensitive response to slight forces.

**Fig. S7. Simulation analysis for the curvature response of the flexoelectret touch sensor under curved deformation.** (**A**) Schematic depiction of the analytical model in the curved geometry of the flexoelectret touch sensor. (**B**-**G**) Simulation results for the output performance in response to curvature. The effect of structure parameters: charge density, *σ*_s_ (**B**); electrode radius, *R*_Electrode_ (**D**); and substrate thickness, *H*_PDMS_ (**F**) on the curves of output voltage, ∆*V* versus curvature. The corresponding ∆*V* variations as a function of *σ*_s_ (**C**), *R*_Electrode_ (**E**), and *H*_PDMS_ (**G**) under different curvatures.

**Note S5. Sensitivity of the flexoelectret touch sensor**

In the experiments, to apply a controlled force or downward displacement (*d*) on the flexoelectret touch sensor, a linear actuator together with a commercial force sensor is vertically pressed on the top surface of the flexoelectret touch sensor, which could precisely control *d* and simultaneously measure the applied force *F*. With this configuration, the relation between the force *F* and the corresponding pressure *P* applied onto the contact area (*S* = 25 mm^2^ ) can be calculated by the formula *P* = *F*/*S*, which means 1 N is equivalent to 40 kPa.

Basically, the sensitivity of this flexoelectret touch sensor presents two linear intervals (**Fig. S8A**). At external force <0.5 N, the sensitivity of the flexoelectret touch sensor is ~353 mV/N (or ~8.8 mV/kPa, *R*² > 0.988); while pressures ranging from 0.5 to 3.5 N, the sensitivity increases to ~835 mV/N (or ~20.9 mV/kPa, *R*² > 0.995). It has proved that the maximum and minimum force detection limit are ~1.2 mN and ~3.5 N, respectively. It should be noted that a limited detectable force is as small as approximately 1.2 mN, which is comparable to the magnitude of a gentle finger touch (on the order of 1 mN), as shown in **Fig. S8B**.

**Fig. S8. Electrical output and sensitivity of the flexoelectret touch sensor.** Δ*V* as a function of force ranged from 0~3.5 N, with a limited detectable force of ~1.2 mN.

**Note S6. The effect of thickness on the sensing performance**

Bending-induced flexoelectricity is the polarization caused by a strain gradient, which is possessed by all dielectric materials. [54] Achieving high-performance flexoelectric devices is associated with outstanding intrinsic flexoelectric coefficients and tremendous strain gradients. Notably, strong flexoelectricity is expected in materials with high-dielectric constants. [25] Following this way, relatively high flexoelectric coefficients are observed in certain ferroelectric materials. [42] However, the realization of large strain gradients under macroscopic conditions is extremely challenging. One possible approach is to utilize thin rigid materials that are able to withstand greater deformability before failure and thus generate abundant strain gradient-modulated local polarization. [55]

Apart from the aforementioned rigid materials, polymers, and soft elastomers are naturally flexible and can undergo significant deformation and tolerate substantial strain gradients. [42] However, they exhibit much lower flexoelectric coefficients (≈10⁻⁸ to 10⁻⁹ C·m⁻¹), which is several orders of magnitude smaller than that for ferroelectric materials. [43] This extremely low flexoelectric coefficient of PDMS means that simply decreasing its thickness has a limited effect on the overall flexoelectric response. Thus, it is necessary to further enhance the flexoelectric coefficient of polymers in order to achieve a strong flexoelectric performance. [46] Wen *et al.* has shown that using the flexoelectric-like response of a flexoelectret is a reliable way to increase the flexoelectric coefficient in silicon rubbers (PDMS) by more than two orders of magnitude. It is also observed in the experiment that the effective flexoelectric coefficient of flexoelectret scales up with the increase in the sample thickness. [44]

We systematically investigated the impact of varying the substrate thickness (*H*_PDMS_) on the output performance of the flexoelectret touch sensor by FEA simulations. As shown in **Fig. S7**, the simulation results indicate that the output voltage ∆*V* is directly proportional to the thickness, which follows the flexoelectric-like response characteristics. In addition, it is worth noting that the thickness of PDMS substrate will modify the mechanical property of the device, which means larger depressed force is needed to keep the same curvature. Therefore, the sensing performance of the flexoelectret touch sensor can be effectively controlled by selecting the appropriate thickness of substrate, while maintaining sensitive response to slight forces (**Note S4**).

In order to reveal the effect of thickness on the device performance more comprehensively, we also fabricated the flexoelectret touch sensor with different thicknesses. The curves of voltage response *∆V* versus force with the thickness of 1 mm, 2 mm, and 5 mm are plotted in **Fig. S9**, indicating that the magnitude of output voltage with the applied force is gradually enhanced as thickness decreases. It can be seen that *∆V* increases approximately linearly with the force for the thicker devices (2 mm and 5 mm); whereas the increasing trend of voltage becomes slower when the force is relatively large for a 1-mm-thick PDMS layer. Since the PDMS layer with the additional thickness inevitably absorbs more deformation from external stimuli, the thinner device could induce a larger bending deformation under a small-force condition due to the weakening deformation suppression effect. [11] Note that *∆V* in response to an increase of force is much more in the low-force region than that in the high-force region, suggesting the decrease of layer thickness could achieve a higher sensitivity but a relatively small response range.

As a functional layer to enhance the effective flexoelectric coefficient of flexoelectret, the appropriate thickness of PDMS layer can effectively control the force transfer process to induce the sufficiently large deformation, while keeping a high sensitivity and excellent reliability to meet the demand of actual sensing applications.

**Fig. S9. Force-generated *∆V* of the flexoelectret touch sensors** **with the PDMS layers at the thicknesses of 1 mm, 2 mm and 5 mm, respectively.**

**Note S7. The frequency characteristics of the flexoelectret touch sensor**

To further verify the reliability of the data and provide additional reference, we fabricated more devices and also tested the frequency-dependent output voltage response using a digital source meter (2636B, Keithley); while the voltage response in **Fig. 3G** was detected by a low-noise preamplifier (SR560, Stanford Research Systems) and a data acquisition card (PCI-6259, National Instruments). As shown in **Fig. S10A** and **B**, the amplitude of output voltage is gradually reduced with decreasing frequency, exhibiting a trend almost similar to **Fig. 3G** (replotted below for convenience, **Fig. S10C**).

As we know, mechanical energy is a ubiquitous ambient source, one of the most common and easily accessible energies in our lives. The frequencies of available mechanical vibration in the environment range from a few Hz (human steps, human heart rate, and the waves of the sea) to a few kHz (the mechanical engine). [48] For self-powered sensing applications, the dominant detection bandwidths are typically aligned with the characteristic frequencies of the ambient mechanical stimuli. Therefore, the flexoelectret touch sensor is capable of generating sufficient output signals at frequencies ≥0.1 Hz, satisfying the needs of self-powered sensing in practical scenarios.

**Fig. R10. Frequency-dependent voltage response characteristics of the flexoelectret touch sensor.**

**Note S8. Comparison of the flexoelectret touch sensor with some other sensors**

**(1) Development of the flexoelectronics and flexoelectret studies**

Flexoelectricity describes an unusual electromechanical phenomenon coupling non-uniform deformation or, in other words, a strain gradient field, and electrical polarizations in all dielectric materials. Recently, interface engineering utilizing flexoelectric polarization has enabled seamless integration between semiconductor devices and external stimuli (strain gradients), leading to a novel electronic regulation mechanism—the flexoelectronic effect. [26]

The schematic diagram in **Fig. S11** illustrates the concept of the flexoelectronics, where the inner-crystal polarization field can be induced in a semiconductor upon an inhomogeneous strain. Thus, the strain-gradient-induced flexoelectric polarization would have a huge impact on the concentration and distribution of free carriers at the interface region, which allows the effective modulation of the interface Schottky barrier height and width, thereby controlling the charge transport. [56]

**Fig. S11. The fundamental principle of the interfacial engineering by local polarization in the flexoelectronics.** The schematic diagram of the modulation on the carrier transport mechanism and the corresponding energy band profile in the metal-semiconductor interface by the flexoelectronic effect.

Since strain gradients usually scale up inversely with the decrease in the sample size, the flexoelectric polarization is found to be size dependent. [46] Previous studies on the flexoelectronics are mainly focus on small length scales, which relies on atomic force microscope (AFM) tips or micrometer-scale probes. Recently, Wang *et al.* demonstrated a giant flexotronic effect in the nanoscale volume of semiconductors (Si, TiO_2_ and Nb-SrTiO_3_), exhibiting a high strain sensitivity with a gauge factor > 2650. [26] Guo *et al.* developed the silicon flexoelectronic transistors (SFTs) and system including two modulation mechanisms (channel width gating and interfacial barrier gating), which can enable tactile sensing for the recognition of force location and show high sensitivity with a gauge factor of ~2189. [27] However, the size effect restricts the scale at which flexoelectronics can be effectively utilized, which suppresses its applications at the macroscale.

Developing a universal strategy to substantially enhance the flexoelectric coefficients at the macroscopic scale remains challenging in the field. [24] An effective approach we pursue in this paper is embedding charges and dipoles into the dielectric material to obtain electrets, which are called flexoelectrets. [57] Wen *et al.* observed experimentally that the flexoelectric coefficient of flexoelectret is two orders of magnitude higher than that in polymers (PDMS), which indicates a possibility of introducing flexoelectricity into the macroscale. [44, 51] The flexoelectret possesses the advantages of high flexibility, biocompatibility and light weight, which makes it a promising material for constructing self-powered flexible electronics. [38]

**(2) Comparison of the performance with existing self-powered sensors**

So far, numerous studies about self-powered sensing have been conducted to detect signals from the environment, such as piezoelectric and triboelectric effects for the perception of mechanical energy. As the reviewer suggested, in order to intuitively exhibit the position of the flexoelectret touch sensor in comparison with other sensors, we have summarized their performance parameters (mechanism, material/structure, sensitivity, detection range, and durability) as listed in the following **Table S1**.

Basically, the pressure sensitivity of this flexoelectret touch sensor presents two linear intervals. At pressures <20 kPa, the sensitivity of the flexoelectret touch sensor is ~8.8 mV/kPa (*R*² > 0.995), while pressures ranging from 20 to 140 kPa, the sensitivity increases to ~20.9 mV/kPa (*R*² > 0.995). It has proved that the maximum and minimum pressure detection limit are 0.047 and 140 kPa, respectively. As show in **Table S1**, the sensitivity of the prepared flexoelectret touch sensor is in the middle level and has a large detection range.

Furthermore, it is worth noting that some piezoelectret and triboelectric sensors can achieve large output voltage and thus high sensitivity. [49, 58, 59] However, these devices are often vulnerable to environmental disturbances, such as variations in temperature and humidity.[20] Moreover, the high sensitivity is generally exhibited in the low-pressure region, and the sensitivity would drop dramatically as the pressure increases. In contrast, the flexoelectret touch sensor exhibits stable performance against external interferences, considering that the device is entirely encapsulated in the protective PDMS layers. More importantly, its sensitivity tends to increase under large mechanical deformation, thereby complementing conventional piezoelectric and triboelectric sensing in the high-pressure region. Therefore, the flexoelectret sensor offers a potential strategy for developing hybrid structured devices with ultrahigh sensitivity over the full measurement scale. [60]

**Table S1. Summary of sensitivity of self-powered pressure sensors**

| Sensing mechanism | Material/  structure | Sensitivity (mV/kPa) | Detection range | Durability (cycles) | Ref. |
| --- | --- | --- | --- | --- | --- |
| Piezoelectric | Dual-layer PVDF films | 7.7 | 80~750 kPa | 80000 | [61] |
| Piezoelectric | P(VDF-TrFE) film | 640 | 0.1~5 kPa | 3000 | [62] |
|  |  | 56 | 5~20.3 kPa |  |  |
| Piezoelectric | Ag/BTO/Ag | 44 | 15.4~27.6 kPa | — | [63] |
| Piezoelectret | THV/COC | 30 | 1~150 kPa | 10000 | [49] |
| Piezoelectret | EVA/FEP | 18980 | 0.5~1 kPa | 5000 | [64] |
|  |  | 770 | 1.6~10 kPa |  |  |
|  |  | 250 | 10~40 kPa |  |  |
| Triboelectric | CA/PVDF-PVA NFs | 480 | 0.1~0.5 kPa | 10000 | [59] |
|  |  | 250 | 0.5~30 kPa |  |  |
|  |  | 170 | 30~135 kPa |  |  |
| Triboelectric | TPU/Ag NWs NF networks | 9973 | 0~1.6 kPa | 40000 | [65] |
|  |  | 538 | 1.6~7 kPa |  |  |
| Triboelectric | PDMS/BiTO-rGO-PVDF | 5070 | 0.2~1.72 kPa | 20000 | [66] |
|  |  | 1890 | 1.72~3.65 kPa |  |  |
| Triboelectric | PTFE/PET film | 45700 | 0~0.71 kPa | 40000 | [67] |
|  |  | 10600 | 0.71~1.3 kPa |  |  |
| Triboelectric | ZZ-MSCA/FEP | 12100 | 1.3~5.2 kPa | 2000 | [68] |
|  |  | 1900 | 5.2~20.9 kPa |  |  |
| Triboelectric | CR-CAPG aerogel/PTFE | 133 | 2.4~28.12 kPa | 7200 | [69] |
|  |  | 34 | 28.12~398.96 kPa |  |  |
| Triboelectric | silk fibroin nanofiber/PTFE | 1767 | 0~25 kPa | 2000 | [70] |
|  |  | 315 | 25~40 kPa |  |  |
| Piezo-triboelectric | Mo/AlN/Mo;  Parylene/PDMS-Ecoflex | 59.4 | 10~50 kPa | 500 | [58] |
|  |  | 160 | 50~120 kPa |  |  |
|  |  | 3.7 | 120~400 kPa |  |  |
| Piezo-triboelectric | PVDF/MWCNT fibrous;  Porous PDMS | 54.37 | 0~80 kPa | 14000 | [60] |
|  |  | 9.80 | 80~240 kPa |  |  |
| Flexoelectret | PDMS/PTFE film | 8.8 | 0~20 kPa | >8000 | This work |
|  |  | 20.9 | 20~140 kPa |  |  |

**Note S9. More anti-interference experiments of the flexoelectret touch sensor**

Further experiments are carried out to demonstrate humidity stability of the flexoelectret touch sensor, the output voltages under different humidity is also investigated (**Fig. S12A**). Since the whole device is encapsulated by PDMS, the flexoelectret touch sensor could maintain reliable sensing performance in a high humidity environment. [71] As the relative humidity increases, no apparent variation of the output voltage signals is observed, as shown in **Fig. S12B**. It is worth noting that when the relative humidity reaches 85%, the output voltage suffers from more noise signals. The result could be attributed to that the electrical connections of the measurement system were exposed to the external environment.

**Fig. S12. Humidity stability of the flexoelectret touch sensor.** (**A**) The output voltage Δ*V* of the flexoelectret touch sensor at different relative humidity (25-85%). (**B**) The corresponding Δ*V* versus humidity.

As on-skin applications, washability is very important for long-term reliability and repeated usage. [65] To evaluate the washability of the flexoelectret touch sensor, the output voltages before and after washing are examined, as shown in **Fig. S13**. It can be found that no apparent variation of the output voltage is observed throughout the washing test, due to the waterproofing characteristics of the PDMS over the conductive components. Considering the constant voltage output against washing, the flexoelectret touch sensor is the potential for widespread application in daily life.

In experiment, a hyper-elastic material (PDMS), which enables large deformation, is utilized to fabricate the flexoelectret touch sensor. To further investigate the mechanical stability, the flexoelectret touch sensor is repeatedly bent for 10000 cycles, and there is no visible performance degradation before and after operating cycles (Fig. S14). As we all know, PDMS exhibits excellent mechanical stability attributing to its intrinsic flexibility, therefore the whole device will not be easily damaged by bending from external force. [60]

These experimental results reveal the excellent anti-interference ability of the flexoelectret touch sensor in the complex environment, which strongly support its potential for practical wearable electronics.

**Fig. S13. Washability of the flexoelectret touch sensor.** (**A**) The output voltage Δ*V* of the flexoelectret touch sensor before and after washing (30 min). (**B**) The photograph of showing the washing test.

**Fig. S14. Stability of the flexoelectret touch sensor** **under repeated bending.** (**A**) The output voltage Δ*V* of the flexoelectret touch sensor before and after 10000 bending cycles. (**B**) The pictures of the flexoelectret touch sensor with application of bending deformation.

**Note S10. Self-calibrated tactile imaging for the touch point localization**

Through further data analysis on the strain interference signals from adjacent pixels, more information could be obtained about local deformation spreading to the surrounding regions [14]. To quantitatively describes the underlying relationship between the relative strain interference signal of adjacent pixel (the output voltage, ∆*V*_Adjacent pixel_ is normalized by the voltage response at touch pixel, ∆*V*_Touch pixel_) and the corresponding distance from the touch pixel (*l*), the fitting function of strain interference distribution (as shown in the inset of **Fig. 6B**) is introduced as follows:

$$\begin{aligned} \frac{\Delta V_{Adjacent pixel}}{\Delta V_{Touch pixel}}=\frac{1}{1+\left( \frac{l}{0.28126} \right)^{1.72528}}\#\text{(3)} \end{aligned}$$

According to the measured strain interference signals along the *x*-axis and *y*-axis of the touch pixel (corresponding to the row and column in the imaging array respectively), the coordinate offset of touch point would be rectified by the statistical characteristic function. As shown in **Fig. 6C** and **Fig. S15**, more accurate position of the touch point could be predicted with a sub-pixel precision. Furthermore, the data values of the adjacent pixels can be filtered to realize the denoise process, using the characteristic function based on the strain interference distribution model. The self-calibrating process can effectively improve the imaging quality beyond the limit of pixel size, which is adapted to the high-resolution tactile imaging for next-generation electronic skin.

To provide a more comprehensive validation of the self-calibration tactile imaging, we have fabricated a 5×5 flexoelectret touch sensor array to provide the additional examples. Through systematic statistical analysis of the strain interference signals from adjacent pixels, we derived a fitting function for self-calibrated touch point localization, as shown in **Fig. S16**.

These additional results from the 5×5 array device further demonstrate that the current self-calibration strategy is primarily applicable to single-point tactile sensing. Reducing the number of pixels would limit the available local deformation information, which may compromise the accuracy of touch point localization.

For more complex deformation involving non-uniform distribution characteristics, different types of signal decoupling are indispensable for self-calibration process. One possible approach is to employ deep learning techniques or other data-driven algorithms, thereby enhancing accuracy and adaptability to multi-modal sensing applications. [72-74] However, its complexity requires lots of further in-depth investigations.

**Fig. S15. Localizing the touch point based on the strain interference distribution characteristics for self-calibrated tactile imaging.** The intersections of the dotted lines denote the estimated positions of touch points via self-calibrated localization in the *x*-axis and *y*-axis directions, corresponding to the pixels from (1,1) to (7,7) along the diagonal in **Fig. 5D**.

**Fig. S16. Self-calibrated tactile imaging for touch point localization using a 5×5 flexoelectret touch sensor array.**

**Note S11. Scalability of the flexoelectret touch sensor to high-resolution array**

The flexoelectret transduction mechanism is inherently adapted to scalability. The self-powered response originates from the touch-induced curved geometry that generates non-symmetric electric field, coupling the flexoelectric-like effect and electrostatic induction. The fabrication approach of the patterned electrodes allows the pixel dimensions to be adjusted independently of the active material. Consequently, the larger-area and high-resolution array can be achieved by increasing the electrode density, while remaining the structure of the charged electret layer unchanged.

The present study demonstrates a 7×7-pixel array prototype for self-powered tactile imaging. A two-electrode structure is disposed on the top and bottom surfaces with a wiring count proportional to n×m, which ensures complete electrical isolation between adjacent pixels and effectively eliminates crosstalk. For large-area tactile arrays, this two-electrode structure (direct addressing) becomes quite complex for high-resolution applications due to the increased number of wire connection. Lin *et al.* reported a dual-layer piezoelectric sensor array with an alternative electrode topology, which could reduce the wire interconnects to n+m+2 for an n×m sensor array. [61] This multiplexing strategy with the row+column electrodes can be extended to more pixels using a small quantity of wires. Adapting the row+column electrode topology to the flexoelectret sensor array may provide a viable approach to reduce wiring complexity and facilitate large-area scalable fabrication. However, the integration with the flexoelectret transduction mechanism requires further in-depth investigation, in order to address challenges associated with deformation suppression in the multilayer structure.

For the flexoelectret sensor, the amplitude of voltage outputs outputs are relatively large under gentle touch, which will facilitate simplifying the readout electronics. [31] A multiplexed scanning scheme operating on the row+column matrix would minimize the data acquisition channels for wearable systems. [48] These considerations outline a feasible route toward high-resolution scalable tactile imaging arrays, though further engineering development remains necessary.

**Fig. S17. Experimental setup for the sensing performance characterizations.** (**A**) Illustration of the electrical connection methodology for the characterization of current. (**B**) Demonstration of the cyclic loading applied normal to the device surface. The inset shows the top view of the flexoelectret touch sensor device with central electrodes (electrode dimension, 5 mm × 5 mm).
